# Modulation of stromal cell-derived factor 1 alpha (SDF-1α) and its receptor CXCR4 in *Porphyromonas gingivalis-*induced periodontal inflammation

**DOI:** 10.1186/s12903-016-0250-8

**Published:** 2016-07-22

**Authors:** Jiang Sun, Eiji Nemoto, Guang Hong, Keiichi Sasaki

**Affiliations:** Department of Periodontics and Oral Mucosa Disease, Dalian Stomatological Hospital, 935 Changjiang Road, Shahekou District Dalian, 116021 China; Department of Periodontology and Endodontology, Tohoku University Graduate School of Dentistry, 4-1 Seiryo-machi, Aoba-ku, Sendai, 980-8575 Japan; Liaison Center for Innovative Dentistry, Tohoku University Graduate School of Dentistry, 4-1 Seiryo-machi, Aoba-ku, Sendai, 980-8575 Japan; Division of Advanced Prosthetic Dentistry, Tohoku University Graduate School of Dentistry, 4-1 Seiryo-machi, Aoba-ku, Sendai, 980-8575 Japan

**Keywords:** Fibroblasts, SDF-1α, CXCR4, *P. gingivalis*, Periodontal inflammation

## Abstract

**Background:**

The production of chemokines by tissue resident cells during inflammation is considered one of the main mechanisms involved in the formation of inflammatory infiltrates. Fibroblasts are the main resident cell type in gingival and periodontal ligament tissues, and their ability to produce chemokine stromal cell-derived factor 1 alpha (SDF-1α) and its receptor CXCR4 under stimulation by gram negative bacteria, *Porphyromonas gingivalis,* commonly found in periodontal infections was investigated.

**Methods:**

Western blots were used to assess SDF-1α and CXCR4 protein expression levels in human gingival fibroblast cells (HGF-1) induced by Lipopolysaccharide (LPS) from *P. gingivalis* in the presence or absence of LY294002, a highly selective inhibitor of PI-3K/Akt. RT-PCR and quantitative Real-time PCR was performed using gingival mRNAs from periodontitis patients. Immunohistochemistry was performed to analyze the expression and subcellular localization of SDF-1α and CXCR4, together with NF-kβ phosphorylation, in specimens from patients with periodontitis and in an experimental rat periodontitis model.

**Results:**

We found that *P. gingivalis* LPS up-regulated SDF-1α and CXCR4 protein levels and elevated phosphorylation of the SDF-1α-responsive NF-kβ and Akt at 24 h in HGF-1 cells. SDF-1α and CXCR4 mRNA and protein expression levels were high in all patients with periodontitis. In the *P. gingivalis*-induced rat experimental periodontitis model, SDF-1α and CXCR4 immunoreactivity was higher in gingival and periodontal ligament tissues compared to the control.

**Conclusion:**

Our data showed that PI-3K/Akt is an upstream participant in the *P. gingivalis* LPS-mediated induction of SDF-1α. Taken together, these results suggest that the chemokine SDF-1α and its receptor CXCR4 contribute to *P. gingivalis*-induced periodontal inflammation.

## Background

Fibroblasts are tissue-resident cells presenting unique phenotypes according to their tissue of origin. Recently, fibroblasts have been suggested to be important sentinel cells in the immune system [1] and their reactions to various microbial stimuli, including Gram-negative bacteria-derived virulence factors, have been studied in diverse situations [2–4]. Fibroblasts actively define the structure of the tissue microenvironment and regulate inflammatory responses by producing cytokines and chemokines [1, 5]. Lipopolysaccharide (LPS) derived from the outer membrane of the Gram-negative bacteria, Porphyromonas gingivalis (*P. gingivalis*), is one of the most commonly investigated virulence factors used to activate the innate immune system [6, 7].

Periodontal pathogens, including *P. gingivalis*, can cause inflammation [8, 9], which results in soft tissue destruction and periodontal bone resorption in the development of periodontal disease. The host-immune response to bacteria has been suggested to be associated with the alteration or even the progression of this disease [10, 11]. A wide variety of cytokines, chemokines and their receptors are synthesized by gingival fibroblasts, epithelial cells, endothelial cells and inflammatory cells [12, 13]. The production of chemokines by those cells may vary significantly when comparing fibroblasts from different tissues [14–16], thus suggesting their important role in inflammatory infiltrate formation and cellular traffic during tissue repair.

The chemokine CXCL12, also known as stromal cell-derived factor 1 alpha (SDF-1α), is constitutively expressed by human gingival fibroblasts (HGFs) and by human periodontal ligament (PDL) fibroblasts (HPDLFs). CXCL12 is responsible for regulating the trafficking of bone marrow progenitor cells, as well as the transendothelial migration of leukocytes [17, 18]. SDF-1α plays a role as an essential and non-redundant factor involved in tissue remodeling, specifically in vascular regeneration [19]. Increased SDF-1α gene expression has also been reported in an experimental model of bacterial-induced apical periodontitis [20, 21]. Individuals with periodontal disease have higher levels of SDF-1α in their gingival crevicular fluid compared to healthy individuals and neutrophil migration is also enhanced in the presence of this chemokine [22]. In addition, SDF-1α from PDL cells was demonstrated to participate in the regeneration and homeostasis of periodontal tissues via the recruitment of stem cells [23]. SDF-1α has been shown to promote cell viability and proliferation, to affect differentiation and to exert a migratory effect on PDL stem cells in vitro [24]. Moreover, SDF-1α is up-regulated after myocardial infarction, bone or cartilage fracture, and ischemic cerebral injury at injured sites [25]. Nevertheless, the involvement of SDF-1α in the progression of periodontal inflammation and its potential interactions with signaling cascades are not yet known.

SDF-1α regulates numerous homeostatic and pathological processes through its receptor CXCR4, by inducing several signaling transduction pathways, including activation of the PI-3K/Akt-NF-kβ axes [26, 27]. Expression of CXCR4 is regulated by NF-kβ and the CXCR4 promoter contains p50/p65 binding sites [26, 28]. Moreover, stimulation of mesoangioblasts with SDF-1α triggers the nuclear translocation of NF-kβ p65, which is required for mesoangioblast migration in response to SDF-1α [29].

Considering the complex reciprocal functional interactions that the pleiotropic chemokine SDF-1α can establish with the periodontal microenvironment, the aim of this study was to evaluate SDF-1α and CXCR4 in fibroblasts of gingiva and of PDL origin in terms of the signaling cascade of PI-3K/Akt and the regulation of NF-kβ. Human gingival fibroblast cells stimulated with LPS from *P. gingivalis* exhibited an increased expression of SDF-1α and CXCR4 via activation of the PI3K/Akt and NF-kβ signaling pathways. By employing the *P. gingivalis*-induced experimental rat periodontitis model, we provide evidence that SDF-1α and CXCR4 proteins play an inflammation-promoting role in the development of periodontitis by regulating the function of NF-kβ.

## Methods

### Cell culture

HGF-1 cells were obtained from the American Type Culture Collection (CRL-2014, ATCC, Manassas, VA, USA) and were cultured in Dulbecco’s modified Eagles medium (DMEM) containing 10 % fetal bovine serum (FBS). HGF-1 cells were seeded in 60-mm plastic tissue culture dishes and incubated in 5 % CO_2_ at 37 °C. When the cells reached sub-confluence, they were harvested and sub-cultured. The cells at the fourth passage were used in the experiments. LPS from *P. gingivalis* was added to the cultures for 24 h to evaluate the effects of treatment with an inflammatory cytokine. Where noted, cells were treated with 10 μM LY294002 (Calbiochem, San Diego, CA. USA) for 1 h prior to LPS treatment. The concentration of LY294002 was adopted from our previous study [30].

### Preparation of bacteria

*P. gingivalis* ATCC 33277 was grown in brain heart infusion broth supplemented with 5 mg/ml yeast extract, 5 μg/ml hemin and 0.2 μg/ml vitamin K_1,_ as described previously [31]. Bacterial cells were grown under anaerobic conditions (85 % N_2_, 10 % H_2_ and 5 % CO_2_) at 37 °C for 24 h. LPS from *P. gingivalis* ATCC 33277 was obtained from *P. gingivalis* according to the manufacturers’ instructions (iNtRON Biotechnology, Kyungki-Do, Korea). In brief, 5 ml of a bacterial cell suspension were centrifuged for 30 s at 13,000 rpm at room temperature to remove all traces of the supernatant, and were then vortexed vigorously with 1 ml lysis buffer. Two hundred μl chloroform were then added and vortexed vigorously for 20 s, and then incubated at room temperature for 5 min. The suspension was then centrifuged at 13,000 rpm for 10 min at 4 °C and then 400 μl of the supernatant was transferred to a new 1.5 ml tube, mixed with 800 μl purification buffer and incubated for 10 min at −20 °C. The sample was then centrifuged at 13,000 rpm for 15 min at 4 °C. After washing the LPS pellet with 1 ml 70 % EtOH, it was dried completely. Seventy μl of 10 mM Tris-HCl buffer (pH 8.0) were added to the LPS pellet and sonicated.

### Experimental periodontitis

An established method of experimental periodontitis has been previously reported [31]. Briefly, twelve 5 week-old male Sprague-Dawley rats (CLEA Japan, Inc., Tokyo, Japan) were given sulfamethoxazole (1 mg/ml) and trimethoprim (200 μg/ml) in their drinking water for 4 days to reduce any existing oral microorganisms, followed by a 3 day antibiotic-free period before starting the oral challenges with bacteria. Rats had free access to laboratory chow and tap water. They were randomly divided into two experimental groups [Group A: 5 % carboxymethylcellulose (CMC) (control group); Group B: *P. gingivalis* ATCC 33277 (*P. g*. group)] of 6 rats each. Each rat infected with *P. gingivalis* received 0.5 ml (1.0 × 10^8^ cells/ml) of the bacterial suspension in 5 % CMC by oral gavage at 8, 10 and 12 days. All rats were sacrificed by CO_2_ inhalation 30 days after the last gavage.

### Immunohistochemistry

In total, 17 formalin-fixed, paraffin-embedded gingival tissues were obtained from chronic periodontitis patients (9 male, 8 female). Gingival tissues were obtained from the individuals undergoing periodontal surgery, showed moderate to severe disease and were classified as a periodontitis group with probing depths >4-6 mm. The diagnostic criteria for periodontal disease were performed according to the American Academy of Periodontology [32]. Plaque index (PLI) and gingival index (GI) were evaluated as proposed by Silness and Loe’s method [33]. Clinical attachment level (CAL) was determined by measuring the distance from the periodontal pocket depth to the cementoenamel junction. Severity is based on the amount of CAL and is designated as moderate (4 mm CAL) or severe (>5 mm CAL). Clinical measurements, including probing of pocket depth (PD), bleeding on probing (BOP) were recorded using a manual probe (PCP-UNC 15, Hu-Friedy Chicago, IL, USA) at six sites per tooth and the reading was recorded to the nearest 1 mm. Formalin-fixed, paraffin-embedded gingival tissue sections were immunostained for SDF-1α, CXCR4, and phospho NF-kβ, with the CSA II System (Dako, Carpinteria, CA, USA), in accordance with the manufacturer’s instructions. Sections were initially immersed in Target Retrieval Solution (DAKO) at 95 °C for 12 min, and then cooled for 30 min. Endogenous peroxidase activity was blocked with REAL Peroxidase-Blocking Solution (S2023, DAKO) for 30 min. Antibodies against SDF-1α (1:50; Abcam, Cambridge, MA, USA), CXCR4 (1:50; Abcam) and phospho NF-kβ (1:100; Bioss, Inc., Woburn, MA, USA), were used as primary antibodies and were incubated overnight at 4 °C. The secondary antibodies conjugated to peroxidase (Nichirei Biosciences, Tokyo, Japan) were incubated at room temperature for 30 min. After rinsing with PBS, all specimens were color developed with a 3,3′-Diaminobenzidine tetrahydrochloride (DAB) chromogen kit (Dako), counterstained with hematoxylin, and examined by light microscopy. The immunostaining of all specimens was performed simultaneously to ensure the same antibody reaction and DAB exposure conditions.

### RNA extraction, RT-PCR and quantitative RT-PCR analysis

Total RNA was extracted from gingival tissues using an RNeasy Mini Kit (Qiagen, Tokyo, Japan) and reverse-transcribed using High Capacity RNA-to-cDNA Master Mix (Applied Biosystems, Foster City, CA, USA). For RT-PCR, the reaction mixture (20 μL) contained 1 μL of diluted cDNA sample and 10 pmol of each pair of oligonucleotide primers. PCR conditions included an initial denaturation at 95 °C for 10 min, followed by a 30-cycle amplification consisting of denaturation at 94 °C for 15 s, annealing at 55 °C for 30 s and extension at 72 °C for 30 s. The primers used in RT-PCR analysis are as follows: SDF-1 (5′-agagccaacgtcaagcatct-3′ Forward, 5′-gggcagcctttctcttcttc-3′ Reverse); CXCR4 (5′-ctgagaagcatgacggacaa-3′ Forward, 5′-tcgatgctgatcccaatgta-3′ Reverse); β-actin (5′-agccatgtacgttgcta-3′ Forward, 5′-agtccgcctagaagca-3′ Reverse). All primer pairs were checked for primer-dimer formation using the three-step protocol described above without the addition of the RNA template. For the standard PCR, the products were separated on 1.5 % agarose gels and visualized by ethidium bromide staining. The relative expression levels of target mRNAs, compared to the level of β-actin RNA, were analyzed by real time PCR with the corresponding TaqMan MGB probes (Hs03676656_mH for SDF-1α, Hs00607978_s1 for CXCR4, and Hs99999903_m1 for β-actin) using QuantStudio 6 Real Time PCR System (Applied Biosystems). The thermal cycling conditions were according to the TaqMan Fast Universal PCR protocol.

### Western blot analysis

HGF-1 cells were harvested in RIPA lysis buffer (Santa Cruz Biotechnology, Santa Cruz, CA, USA). Samples were boiled for 5 min, chilled on ice for 5 min, and centrifuged. Equal amounts of protein (20 μg) were electrophoresed on sodium dodecyl sulfate–polyacrylamide gel electrophoresis (SDS-PAGE) gels and electrophoretically transferred to nitrocellulose membranes (Bio-Rad, Hercules, CA, USA). The membranes were blocked with the blocking solution in TBS containing 0.01 % Tween 20 to reduce nonspecific binding, probed overnight at 4 °C with the following primary antibodies: anti-SDF-1 rabbit polyclonal antibody (1:500, Abcam, Cambridge, MA, USA), anti-CXCR4 rabbit monoclonal antibody (1:500, Abcam), anti-Akt rabbit monoclonal antibody (1:1000; Cell Signaling Technology, Beverly, MA, USA), anti-phospho Akt rabbit monoclonal antibody (1:2000; Cell Signaling Technology), anti-NF-kβ p65 rabbit monoclonal antibody (1:1000; Cell Signaling Technology), and anti-phospho NF-kβ p65 rabbit polyclonal antibody (1:1000; Cell Signaling Technology). Anti-β-actin rabbit polyclonal antibody (1:1000; Cell Signaling Technology) was used for loading control. The blots were then incubated for 1 h with a horseradish peroxidase–conjugated anti-rabbit secondary antibody (1:2000; Cell Signaling Technology). Protein bands were developed by ECL plus Western blotting detection reagent (GE Healthcare Bio-Sciences, Pittsburgh, PA, USA) and imaged with an ImageQuant LAS 4000 Mini (GE Healthcare Bio-Sciences). Protein bands were scanned and analyzed by densitometry using the ImageJ software (NIH).

### Statistical analysis

Significant differences were analyzed by Fisher’s exact test. A *P*-value of less than 0.05 is considered statistically significant.

## Results

### Effects of *P. gingivalis* LPS on the expression of SDF-1α and CXCR4 in HGF-1 cells

**Fig. 1:**
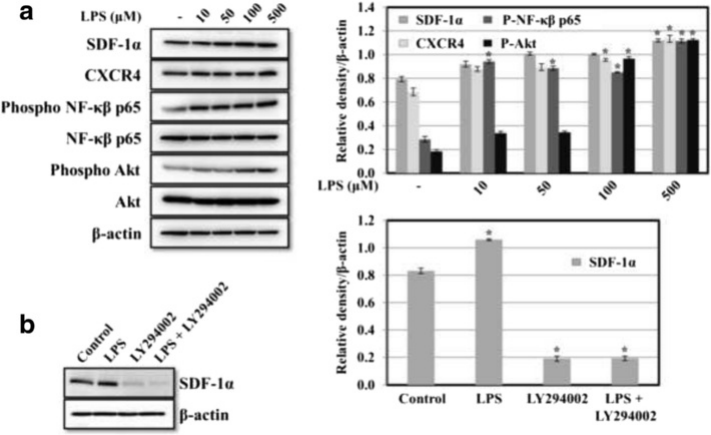
**a** Effect of LPS on the expression of SDF-1α and CXCR4 in HGF-1 cells. HGF-1 cells were incubated with different concentrations of LPS from *P. gingivalis* for 24 h. The cell lysates were then assayed to determine the expression of SDF-1α and CXCR4, Akt and NF-kβ p65, and the phosphorylation of Akt and NF-kβ p65 using Western blots. Membranes were stripped and re-probed with an anti-β-actin antibody as a loading control. Protein bands were quantified by densitometric analyses. The results are expressed as means ± S.D. (*p < 0.05). **b** Role of PI-3K/Akt in *P. gingivalis* LPS-stimulated SDF-1α expression. HGF-1 cells were pre-treated with or without LY294002 for 1 h and were then incubated with or without *P. gingivalis* LPS (500 μM) for 24 h. Cell lysates were assayed using Western blots to determine the expression of SDF-1α. Membranes were stripped and re-probed with an anti-β-actin antibody as a loading control. Protein bands were quantified by densitometric analyses. The results are expressed as means ± S.D. (*p < 0.05)

To understand whether inflammatory gingival activation alters the expression levels of SDF-1α and/or CXCR4, cultures of HGF-1 cells were exposed to *P. gingivalis* LPS at different concentrations, and SDF-1α and CXCR4 protein expression levels were evaluated by western blotting. In line with the cytokine functions of SDF-1α, the data demonstrate that *P. gingivalis* LPS increased SDF-1α and CXCR4 expression levels after 24 h of exposure (Fig. 1a). Moreover, the data in Fig. 1a show that *P. gingivalis* LPS stimulated NF-kβ and Akt phosphorylation in a dose-dependent manner.

### Involvement of PI3-K/Akt in *P. gingivalis* LPS-induced expression of SDF-1α

To determine whether the PI-3K/Akt cascade plays an important role in the LPS-induced expression of SDF-1α in HGF-1 cells, pre-treatment of HGF-1 cells with a pharmacological inhibitor of PI-3K, LY294002, significantly attenuated the *P. gingivalis* LPS-stimulated expression of SDF-1α, suggesting the involvement of PI-3K/Akt in that increased expression (Fig. 1b). The concentration of *P. gingivalis* LPS used (500 μM) was adopted from the results shown in Fig. 1a. A preliminary screening was done to obtain the optimal concentration of LY294002 using the MTS assay and the LDH cytotoxicity assay with *P. gingivalis* LPS-treated cells (data not shown). These results suggest that PI-3K/Akt plays an important role in the *P. gingivalis* LPS-induced expression of SDF-1α in HGF-1 cells.

### Periodontitis stimulates SDF-1α and CXCR4 mRNA in human periodontal tissues

**Fig. 2:**
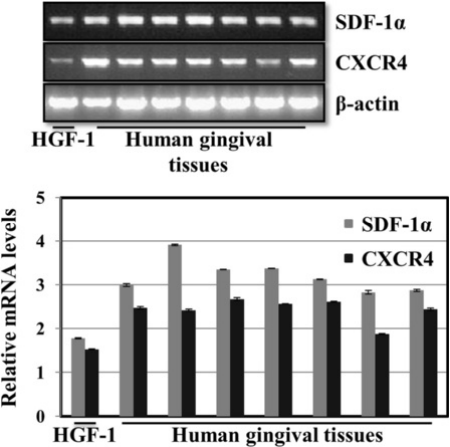
Level of SDF-1α and CXCR4 mRNAs in human periodontal tissues. Total RNA was isolated from each sample and was subjected to RT-PCR and quantitative real-time PCR analysis. SDF-1α and CXCR4 mRNAs were highly expressed in human periodontitis tissues. Relative mRNA levels were calculated as a ratio to the housekeeping gene (β-actin). Each bar represents the mean ± SD for at least 3 independent experiments. HGF-1 cells were used as a positive control

In order to confirm the expression levels of SDF-1α and CXCR4 in human periodontal tissues, their expression was evaluated using RT-PCR and quantitative real-time PCR. HGF-1 cells were used as a positive control. SDF-1α and CXCR4 mRNAs were identified in HGF-1 cells and in tissue homogenates from human periodontal tissues (Fig. 2).

### Increased SDF-1α and CXCR4 immunostaining in human periodontal tissues

**Fig. 3:**
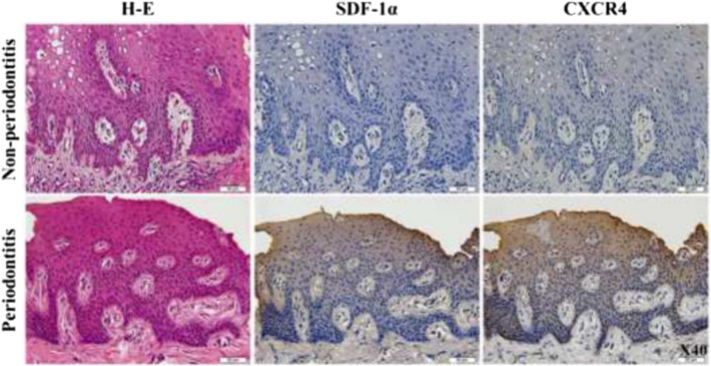
Immunohistochemical analysis of SDF-1α and CXCR4 in periodontal inflammation. Four μm thick sections of formalin-fixed, paraffin-embedded specimens were deparaffinized and immunoreactivity was detected using a DAKO ENVISION Kit. SDF-1α and CXCR4 was highly expressed in patients with periodontitis. SDF-1α and CXCR4 expression in human periodontal tissues was present predominantly in the granular and spinous layers of epithelial cells, while their expression was faint in nonperiodontitis tissues. SDF-1α and CXCR4 expression was also present in the suprabasal layer of epithelial cells. Scale bars, 50 μM

In chronic inflammatory conditions, SDF-1α and CXCR4 expression was observed in suprabasal layers of the epithelium of periodontitis patients. There was also positive staining for SDF-1α and CXCR4 present in some nuclei in the basal layer (Fig. 3). Faint expression of SDF-1α and CXCR4 protein was found in the epithelium of nonperiodontitis controls.

### *P. gingivalis*-induced gingival inflammation triggers activation of the SDF-1α/NF-kβ signaling pathway

**Fig. 4:**
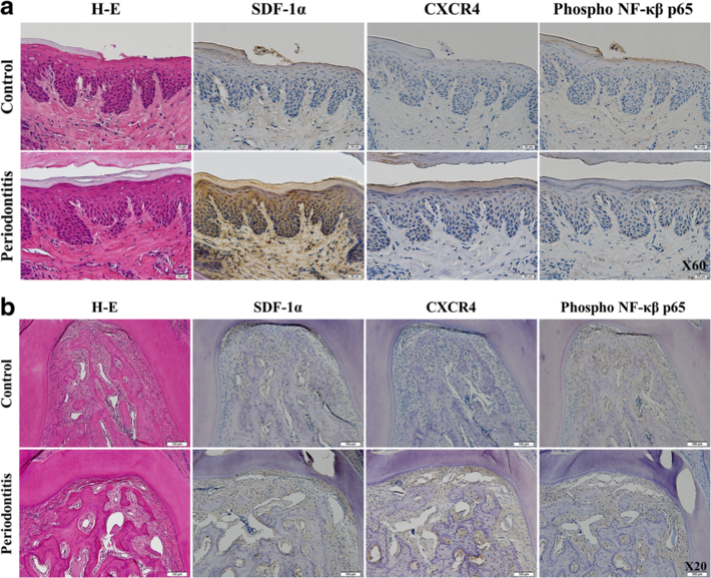
Immunohistochemical analysis of SDF-1α and CXCR4 in *P. gingivalis* challenged experimental rat periodontal inflammation. SDF-1α and CXCR4 were abundantly expressed in the *P. gingivalis* challenged rat tissues. Immunohistochemical analysis revealed a higher expression of SDF-1α and CXCR4 in *P. gingivalis* challenged rat gingiva (Fig. 4a; Scale bars, 20 μM) and PDL (Fig. 4b; Scale bars, 100 μM) compared to the control

SDF-1α and CXCR4 immunohistochemistry revealed a strong staining in *P. gingivalis* challenged gingival epithelium compared to the control (Fig. 4a). SDF-1α and CXCR4 were only weakly expressed in the gingiva of control animals. A strong reaction with anti-SDF-1α and anti-CXCR4 antibodies was found in *P. gingivalis* challenged PDL cells (Fig. 4b). Analysis of the immunostaining pattern of NF-kβ phosphorylation in the experimental rat periodontitis model revealed that it was mainly localized to inflammatory infiltrates in PDL cells (Fig. 4b), whereas no detection was observed in control PDL cells.

## Discussion

In this study, an up-regulation in SDF-1α protein expression by HGF-1 cells was detected in a concentration-dependent manner when they were stimulated with LPS from *P. gingivalis*. The induction of phospho NF-kβ protein expression when cells were challenged by LPS was significant for HGF-1 cells in vitro and PDL cells in vivo, highlighting the important participation of this molecule in inflammatory events of those tissues. We detected an increase in SDF-1α and CXCR4 expression by HGF-1 cells starting at 6 h (data not shown), which was maintained at 24 h. We observed a trend of increased expression depending on the LPS concentration in HGF-1 cells. Moreover, *P. gingivalis* is an important factor of SDF-1α/CXCR4 expression by HGFs in periodontal tissue, and the increase of SDF-1α and CXCR4 expression might be related to the progression of periodontal disease. Taking this into account, it is possible that the induction of SDF-1α expression contributes to the inflammation because, as documented in this study, the expression of signaling molecules, such as NF-kβ, seems to occur. Another study [22] demonstrated that SDF-1α levels were altered due to periodontal inflammation, thus corroborating our speculation regarding the participation of SDF-1α as an important chemokine in the inflammatory microenvironment context. The unveiling of the underlying molecular pathways regulating the inflammatory destruction of periodontal tissue during the progression of periodontitis could provide new and refined therapeutic approaches to prevent tooth loss.

The magnitude of HGF responses to LPS from *P. gingivalis* varied markedly between individuals, and in particular, it is possible that the heterogeneity in these responses was caused by the variability in the host’s genetic background of these cells [34]. Fedyk et al. [35] and Hosokawa et al. [7] demonstrated a decrease in SDF-1α in primary gingival fibroblasts under proinflammatory conditions. In contrast, Jiang et al. [36] showed the up-regulation of SDF-1α and its receptor CXCR4 in inflamed dental pulps. CXCR4 gene expression in periapical tissues was shown to be up-regulated after endodontic intervention in necrotic teeth [37], suggesting an important role of SDF-1α in tissue repair. On the basis of these data, it seems reasonable to assume that SDF-1α expression by fibroblasts is directly dependent on the tissue origin and bacterial product nature.

Considering that SDF-1α is an important chemoattractant for hematopoietic and mesenchymal stem cells supporting their survival and proliferation [38], and that this mediator exerts a migratory effect on PDL stem cells in vitro [24], it seems important to know that by knocking down Akt it would be possible to decrease crucial proinflammatory mediators, affecting chemokines involved in the repair process, such as SDF-1α. A PI-3K inhibitor, LY2940002, results in the inhibition of SDF-1α protein expression in this study. The current results indicate that the PI-3K/Akt pathway may relate to the LPS-induced expression of SDF-1α in HGF-1 cells. Signaling through the innate immune system by fibroblasts, the most numerous resident non-professional immune cells in the periodontium, controls the secretion of important cytokines that modulate the inflammatory microenvironment.

SDF-1α/CXCR4 signaling may have a dual role in inflammation and tissue repair. Participants with periodontal diseases have higher levels of SDF-1α and CXCR4 compared to healthy participants [22], and SDF-1α may recruit host-defensive cells as well as PDL cells to sites of inflammation to be involved in immune surveillance, wound healing and tissue repair. Similarly, expression of both SDF-1α and its receptor CXCR4 has been identified in human dental pulp cells and in stem cells, where those factors have been proposed to participate in the recruitment of cells to the sites of injury [36, 39]. PDL cells are thought to be responsible for the homeostasis and regeneration of periodontal tissues [40–42] following injury or inflammation by bacteria. Therefore, animal studies should be performed to elucidate the in vivo effects of SDF-1α on human PDL cells as well as on inflammatory cells. Our in vivo study detected abundant intracellular SDF-1α and its receptor CXCR4, which was also present on the cell membrane in a small fraction of PDL cells.

The up-regulated expression of SDF-1α at inflammation sites serves as a potent chemoattractant to recruit circulating or residing CXCR4-expressing cells to lesions, with high differentiation and proliferation potential to act as candidates to repair and regenerate the damaged tissues. The interaction of SDF-1α/CXCR4 may play an essential role in promoting the migration of CXCR4-expressing cells into damaged sites during tissue repair. The distinct ability of gingival and PDL fibroblasts to secrete SDF-1α and CXCR4 emphasizes that these cells may similarly contribute to the balance of cytokines in the LPS-challenged periodontium. Considering the importance of resident cells in leukocyte recruitment during local inflammation [43], understanding cellular protection mechanisms against bacterial products may significantly enhance our knowledge about the establishment of periodontal diseases. Our results suggest a potential strategy for in vivo therapies using SDF-1α to promote periodontal regeneration.

## Conclusions

In conclusion, our results demonstrate that levels of SDF-1α and CXCR4 are up-regulated in periodontal inflammation. Since LPS from *P. gingivalis* stimulation increased NF-kβ and Akt activity, they may exert an influence on the levels of SDF-1α and CXCR4. Fibroblasts represent the major SDF-1α expressing cells, which suggest that this chemokine play an important role for sustained immune cell infiltration in periodontitis, particularly of fibroblasts.

## Abbreviations

BOP, bleeding on probing; CAL, clinical attachment level; CMC, carboxymethylcellulose; DAB, 3,3′-Diaminobenzidine tetrahydrochloride; DMEM, Dulbecco’s modified Eagles medium; FBS, fetal bovine serum; GI, gingival index; HGFs, human gingival fibroblasts; HPDLFs, human periodontal ligament fibroblasts; LPS, Lipopolysaccharide; *P. gingivalis*, *Porphyromonas gingivalis*; PD, probing of pocket depth; PDL, human periodontal ligament; PLI, plaque index; SDF-1α, stromal cell-derived factor 1 alpha; SDS-PAGE, sodium dodecyl sulfate–polyacrylamide gel electrophoresis
